# Ultrafast adiabatic quantum algorithm for the NP-complete exact cover problem

**DOI:** 10.1038/srep22307

**Published:** 2016-02-29

**Authors:** Hefeng Wang, Lian-Ao Wu

**Affiliations:** 1 Department of Applied Physics, Xi’an Jiaotong University, Xi’an 710049, China; 2 Key Laboratory of Quantum Information and Quantum Optoelectronic Devices, Shaanxi Province, China; 3 Department of Theoretical Physics and History of Science, The Basque Country University (EHU/UPV), P. O. Box 644, 48080 Bilbao, Spain; 4 IKERBASQUE, Basque Foundation for Science, 48011 Bilbao, Spain

## Abstract

An adiabatic quantum algorithm may lose quantumness such as quantum coherence entirely in its long runtime, and consequently the expected quantum speedup of the algorithm does not show up. Here we present a general ultrafast adiabatic quantum algorithm. We show that by applying a sequence of fast random or regular signals during evolution, the runtime can be reduced substantially, whereas advantages of the adiabatic algorithm remain intact. We also propose a *randomized* Trotter formula and show that the driving Hamiltonian and the proposed sequence of fast signals can be implemented simultaneously. We illustrate the algorithm by solving the NP-complete 3-bit exact cover problem (EC3), where NP stands for nondeterministic polynomial time, and put forward an approach to implementing the problem with trapped ions.

The adiabatic principle addresses that a quantum system governed by a slowly-varying Hamiltonian will remain near instantaneous ground state of the driving Hamiltonian^1^,^2^. It has a variety of applications in quantum information processing, such as adiabatic quantum computing (AQC)^3^, fault-tolerance against specific errors^4^, and universal holonomic quantum computation^5^,^6^,^7^ based on the Berry’s phase^8^,^9^,^10^.

Adiabatic quantum computing is one of quantum computing models that have potential in solving certain problems much faster than their classical counterparts, in particular factoring large integers^11^, searching unsorted database^12^ and simulating quantum many-body problems^13^. AQC is based on the adiabatic principle. The eigenstate of the final Hamiltonian encodes solution to the problem of interest. The runtime of AQC has to be slow to guarantee that the final state is able to reach the ground state of the final problem Hamiltonian. This requires long coherence time in experimental implementation of the process, especially for practical large scale systems. As such, the runtime is crucial for AQC to be valid. If the runtime is too long, quantumness may become vanishingly small due to decoherence and consequently the quantum speedup over classical computation will fade away. Recently an experiment^14^ has been performed to address this crucial question: whether or not a large-scale quantum device has the potential to outperform its classical counterpart? The experimental test was done for finding the ground state of an Ising spin glass model on the 503-qubit D-Wave Two system which are designed to be a physical realization of quantum annealing using superconducting flux qubits. Unfortunately, there was no evidence found for quantum speedup. The main reason for this dysfunction is that the runtime is so long that before the end of an adiabatic quantum algorithm, decoherence has completely ruined all quantumness. Therefore speedup of adiabatic algorithms is crucial in realization of practical large scale quantum computation.

In this paper, we present a general approach that speeds up adiabatic algorithms substantially by applying fast signals during the dynamical evolution process. The proposed protocol is experimentally accessible in a variety of promising quantum-computing setups. We demonstrate this approach by solving a 3-bit exact cover problem (EC3).

## Results

### The Algorithm

The EC3 problem is a particular instance of satisfiability problem and is one of the NP-complete problems. No efficient classical algorithm has been found for solving this problem. On a quantum computer the EC3 problem can be formulated as follows^3^,^15^: for a Boolean formula with *M* clauses


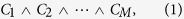


where each clause *C*_*l*_ is true or false depending on the values of a subset of the *n* bits, and each clause contains three bits. The clause is true if and only if one of the three bits is 1 and the other two are 0. The task is to determine whether one (or more) of the 2^*n*^ assignments satisfies all of the clauses, and find the assignment(s) if it exists.

In refs 3,15, a quantum adiabatic algorithm for solving the EC3 problem has been proposed. In this algorithm, the time-dependent evolution Hamiltonian *H*_0_(*t*) is


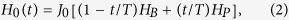


where *H*_*B*_ is the initial Hamiltonian whose ground state is used as the initial state, *H*_*P*_ is the Hamiltonian of the EC3 problem whose ground state is the solution to the EC3 problem and *T* is the total evolution time or the runtime. Here *J*_0_ is the strength of the Hamiltonian and is set as *J*_0_ = 1 in this paper. In this algorithm, the Hamiltonian of the system evolves adiabatically from *H*_*B*_ to the problem Hamiltonian *H*_*P*_, meaning that the system evolves from the ground state of *H*_*B*_ to the ground state of *H*_*P*_. *H*_*B*_ is defined as


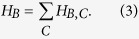


where *H*_*B*,*C*_ is the Hamiltonian of clause *C*. Let *i*_*C*_, *j*_*C*_ and *k*_*C*_ be the 3 bits associated with clause *C*. *H*_*B*,*C*_ is defined as


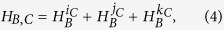


with


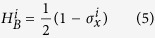


and 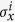 are the Pauli matrices. The Hamiltonian *H*_*P*_ for the EC3 problems is defined as follows: for each clause *C*, one can define an “energy” function


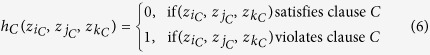


such that


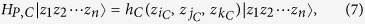


where 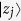 is the *j*-th bit and has a value 0 or 1. Define


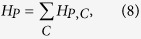


and we then have 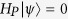, if and only if 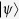 is in a superposition of states 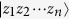, where the bit string 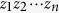 satisfies all of the clauses.

In what follows we will describe our approach for solving the EC3 problem by applying a sequence of fast signals during the dynamical process^16^. We consider a Hamiltonian – a *dressed H*_0_(*t*),


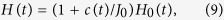


where *c*(*t*) represents a sequence of fast signals. Ref. 16 shows that the adiabaticity can be enhanced and even induced by *c*(*t*)/*J*_0_ – regular, random, and even noisy fast signals. Specifically, *c*(*t*)/*J*_0_ could be a white noise signal in magnetic field, as exemplified in ref. 16. We will use this strategy to speed up adiabatic quantum algorithms and then illustrate our general approach by an experimentally feasible example.

We now come to explain the principle and experimental implementation of our approach in terms of a simple but nontrivial EC3 problem. Consider a 4-bit EC3 problem, where we select the 3-bit set of clauses as {1, 2, 3}, {2, 3, 4}, and {1, 2, 4}. The solution to this problem is 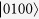.

**Figure 1:**
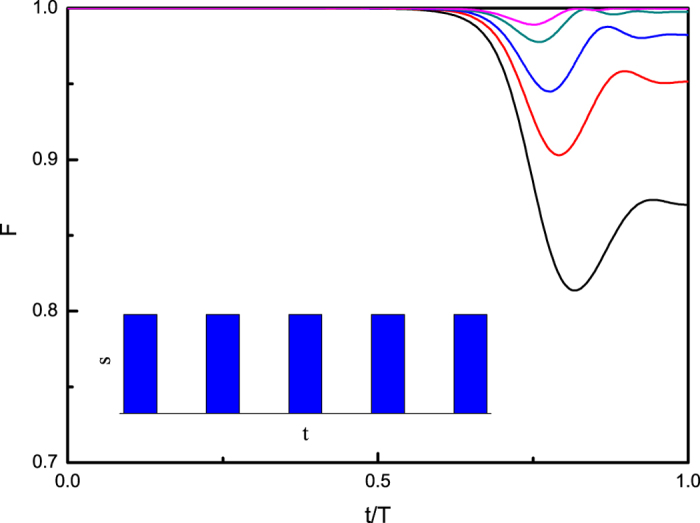
Dynamics of fidelity 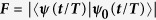. The total evolution time or runtime *T* = 40, the intervals between pulses are set as Δ = 0.08. The black solid curve shows the result for the strength *s* = 0; the red dashed curve for *s* = 0.5; the blue dotted curve for *s* = 1.0 and the dash-dot dark cyan curve shows for *s* = 2.0. The magenta short dashed curve shows the result of the total evolution time *T*_0_ = 160 when the system *enters* the adiabatic regime justified by *F*(*T*) ≥ 0.999. For this model we consider this curve as a reference: *paths* 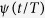 are in the adiabatic regime if *F*(*T*) ≥ 0.999.

For this specific model, we show numerically that when *T*_0_ > 160, the system enters the adiabatic regime. In order to study the contributions of fast signals, we set *T* = 40 < *T*_0_ in the non-adiabatic regime, and apply a sequence of fast regular pulses during the adiabatic process. The pulse strengths are *s* = 0, 0.5, 1.0, 2.0, respectively. Figure 1 shows the dynamics of fidelity 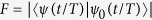 between the system wave function and the instantaneous ground state of *H*(*t*), where 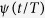 is the wave function governed by the Schrödinger equation or the time-ordering evolution operator and 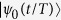 represents the instantaneous ground state of the Hamiltonian *H*(*t*). It is clear in the figure that as the strength of pulses increases, the adiabaticity is induced from a non-adiabatic regime and the fidelity *F* is approaching one, in particular in the region where the solution is encoded. The quality of pulse control can also be improved by increasing the density of fast signals.

**Figure 2:**
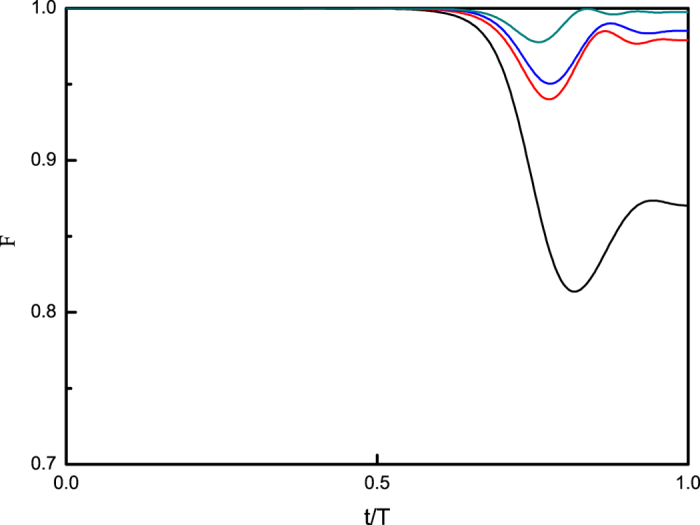
*T* and Δ are the same as in (**a**). The black solid curve shows the dynamics of *F* for the case where no signal is applied; the red dashed curve (the blue dotted curve) shows the fidelity dynamics when applying fast signals 2cos^2^(10*t*) (2sin^2^(10*t*)), and the dark cyan dash dot curve shows *F*(*t*) controlled by fast pulse signals with the pulse strength *s* = 2.0.

**Figure 3:**
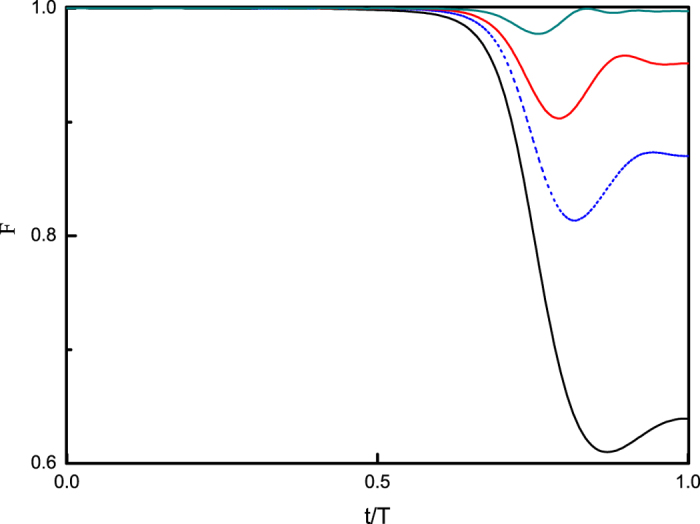
Dynamics of fidelity *F* with the runtime *T* = 20 and the intervals Δ = 0.04. The black solid curve shows the dynamics for *s* = 0; the blue dotted curve for *s* = 1.0; the red dashed curve shows the result for *s* = 2.0 and the dark cyan dash dot curve for *s* = 5.0.

Different types of fast signals work as perfect as regular rectangular pulses^17^. Figure 2 shows the fidelity dynamics by applying different fast signals, even random signals as in Fig. 3. The red dashed line shows the result by an even simpler fast signal 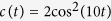 and the blue dotted line is that of 2sin^2^(10*t*). The black solid line uses regular rectangular pulses with *s* = 2.0 and Δ = 0.08, as a reference.

Fast signals reduce the runtime of adiabatic evolution algorithms greatly, and keep very high fidelity *F* particularly when the system reaches the target–the ground state of the problem Hamiltonian *H*_*P*_. Furthermore, the runtime can be even shorten for example to half, *T* = 20. We set the strength of pulses as *s* = 0, 1.0, 2.0, respectively, as in Figure 3. It shows again that the adiabaticity is greatly enhanced even in a shorter runtime by increasing strengths.

Adiabaticity can be induced from an originally very fast dynamical process if pules signals are even stronger. For example, if the signal strength *s* = 15, the system wave function evolves along the adiabatic path in the runtime *T* = 9 and at the very high fidelity *F* = 0.999 overlapping with the eigenstate of *H*_*P*_, which is 17 times faster than the natural adiabatic process where the runtime *T*_0_ = 160. Numerical analysis shows that if we are allowed to increase the strength at will, the runtime *T* can be as fast as we wish. Other examples are, if *s* = 1.0, 5, 30, *T* ≈ 70, 23, 5.0, respectively.

In general, our algorithm can be justified in terms of Leakage Elimination Operators. Consider the instantaneous eigenstates 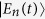 of the Hamiltonian (2). A quantum state at time *t* can then be expressed as


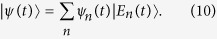


Under the bases 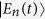, we can rewrite the Schrödinger equation, with the corresponding wave function 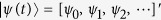 and the *rotating representation* Hamiltonian


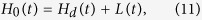


where 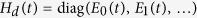 is diagonal and 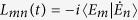 is off-diagonal. Without loss of generality, we set 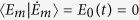, otherwise they can be removed by a simple gauge transformation. Now the reason why we chose the dressed *H*(*t*) in (9) is clear. The *dressing* does not change the off-diagonal *L*(*t*), but only rescales *H*_*d*_(*t*). Ideally, if we turn on the strong and fast control 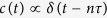 at given times *nτ* 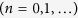, the propagator of the control *c*(*t*)*H*_*d*_(*t*) gives Leakage Elimination Operation (LEO) *R*_*L*_ 18 in the rotating framework, or a rotating LEO. This operator satisfies {*R*_*L*_, *L*} = 0, and serves as a leakage elimination operator: 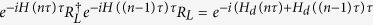 when *τ* → 0 and *t* ≈ *nτ*. This Bang-Bang sequence parity-kicks out the leakage *L*. Furthermore, all leakages such as *LB* can be eliminated by *R*_*L*_, where *B* can be an operator of other system, such as an external bath^18^.

We now illustrate the rotating LEO by a two level system, where the *rotating representation* Hamiltonian reads


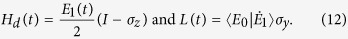


When 
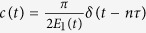
 or parity-kick at *t* = *nτ*, the rotation LEO is *R*_*L*_ = −*iσ*_*z*_, such that *R*_*L*_*L*(*t*)*R*_*L*_ = −*L*(*t*) and *L*(*t*) is parity-kicked out.

The idealization of the Bang-Bang sequence has been proved unnecessary in the recent publications^16^,^17^. The effectiveness of LEOs depends exclusively on the integral of the pulse sequence in the time domain^17^ and the scheme is obviously independent of *n*, the size of the system.

### Randomized Trotter formula and implementation of the algorithm in trapped ions

We now discuss the feasibility to experimentally implement our algorithm on an ion trap quantum information processor. In general, the EC3 problem Hamiltonian is supposedly stored in an Oracle and is called when it is needed. In order to perform experimental demonstration of our algorithm, here we simulate the 4-bit EC3 Hamiltonian with trapped ions. We first write the problem Hamiltonian explicitly in the qubit space,


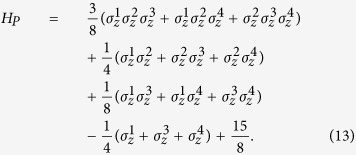


Note that the Hamiltonian contains up to three-body interactions, since symmetry rules out more complicated interactions which may appear in multi-bit EC3 problems.

The time-ordering evolution operators driven by the time dependent Hamiltonians *H*(*t*) and *H*_0_(*t*) cannot be analogously simulated by trapped ions. Therefore digital simulation has to be employed. The standard recipe of digital simulation for adiabatic processes is the use of the Trotter formula, as done in previous literatures^19^. In what follows, we will present a *randomized* Trotter formula (RTF) to mimic *H*(*t*), which effectively combine the two processes, applying fast signals during the dynamics and simulating *H*_0_(*t*).

The time-ordering unitary evolution operator is implemented as


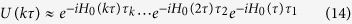


up to order *O*(*τ*^2^). Usually, the evolution operator of *H*_0_(*kτ*) is simulated by setting all *τ*_*j*_ = *τ* where 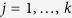.

The distinctive recipe of our RTF is that we set


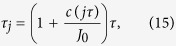


such that 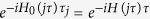. The equality links two different physical operations. The left is the simulated *H*_0_ evolving during a short but uneven time interval *τ*_*j*_, and the right means a fast signal *c*(*jτ*) has been implemented, at the time instance *jτ*, upon *H*_0_ that *transforms* into the dressed *H* evolving in an even time interval *τ*. The mathematical equivalence implies that we can experimentally simulate 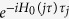 instead of 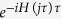, whose simulation ingredient is not yet known (*unknown* for this model but it is simple to implement *c*(*t*) upon *H*_0_ for most systems, such as an additional magnetic fast-varying field upon spins). In other words, the simulation (14) for *H*_0_ becomes that of *H*,


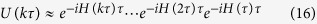


up to order *O*(*τ*^2^).

The evolution operator of *H*_0_ is simulated by the Trotter decomposition,


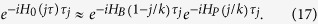


Experimentally, exact control of these uneven time intervals *τ*_*j*_ might not be easy. Therefore, the easiest way for experimentalists is to assign random values to these intervals *τ*_*j*_. This is equivalent to employ random fast signals *c*(*jτ*), which has shown the same excellent control quality as that of other fast signals^16^,^17^.

**Figure 4:**
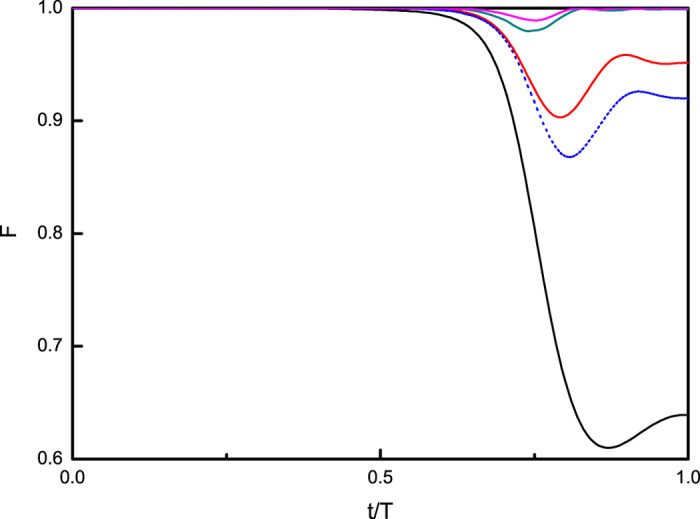
Dynamics of *F*(*t*) with the total evolution time *T* = 20. The black solid curve shows *F* without signal control; the red dashed curve is *F* controlled by a sequence of fast pulses with *s* = 2.0 and Δ = 0.04; the blue dotted and the dark cyan dash dot curves show the dynamics of *F* obtained by the simulated Eq. (16), where *τ*_*j*_ varies randomly in the range [2.0*τ*, 3.0*τ*] and [4.0*τ*, 8.0*τ*], respectively. The magenta short dashed curve is the *T*_0_ = 160 reference for the adiabatic regime.

We set the runtime *T* = 20 and let *τ*_*j*_ change randomly in the range [2.0*τ*, 3.0*τ*] and [4.0*τ*, 8.0*τ*] respectively, and perform simulation. Figure 4 shows the results and compares them with regular pulses. It is clear that random fast signals work as perfect as regular pulses. When the variation range of *τ*_*j*_ is larger, the enhancement of adiabaticity is even better than that of fixed *τ*_*j*_’s, and evolves on the same adiabatic path as that of the adiabatic reference where *T*_0_ = 160.

Now we come to discuss the experimental implementation of the algorithm on trapped ions . It is clear that we need only to implement the slices 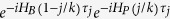 and repeat them to perform the evolution operator *U*(*kτ*). *H*_*B*_ is a simple single-qubit Hamiltonian and can be implemented on most sophistic quantum devices, including trapped ions. It is a challenge for quantum devices to implement three or more body interactions. Fortunately, trapped ions do not have this difficulty. Consider tensor products of Pauli matrices in the form of 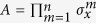. The time evolution operator of *A* can be implemented efficiently with the Mølmer-Sørensen (MS) scheme^20^,^23^ on trapped ions,


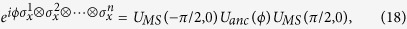


where the exponential is implemented by two MS gates to the *n* system ions and one ancilla qubit (no. 0), 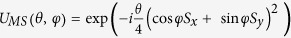, and 
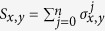
. 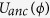 is defined as when *n* is odd, 
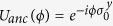
 for *n* = 4*m* + 1, and 
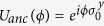
 for *n* = 4*m* − 1, and when *n* is even, 
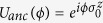
 for *n* = 4*m*, 
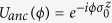
 for *n* = 4*m* − 2. The unitary operator 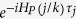 in Eq. (17), which contains tensor products of *σ*_*z*_’s, can be implemented by performing Hadamard transform on each of the *σ*_*x*_ operators in Eq. (18).

In comparison with decomposing the slices of the time evolution operator into single- and two-qubit gates, the number of the gates to be implemented is reduced. This saves resources greatly and helps in the digital implementation of the fault-tolerant quantum computing. In a recent work^21^, trapped ions have been reported that *T*_2_ is of 50 secs and 2000 single qubit gates have been implemented with fidelities significantly above the minimum threshold required for fault-tolerant quantum computing. This is the reason why there have been many quantum simulation proposals using the MS scheme.

## Discussion

A short runtime is of crucial importance for adiabatic quantum algorithms to achieve polynomial time speedups over their classical counterpart, because it is difficult to keep quantumness of a system for long time in presence of noisy environment. In this paper, we propose an adiabatic quantum algorithm assisted with fast signal and show that by applying a sequence of fast signals, the runtime in the adiabatic quantum computing can be greatly reduced^22^. This technique has practical interest in the physical implementation of adiabatic quantum algorithms^23^. We applied this approach to solve the EC3 problem and discuss the feasibility to implement it on trapped ions Fig. 4. We introduce a randomized Trotter formula which effectively implements effects of fast signals upon the original Hamiltonian, which, as we show, can be implemented efficiently on a trapped ion system.

## Additional Information

**How to cite this article**: Wang, H. and Wu, L.-A. Ultrafast adiabatic quantum algorithm for the NP-complete exact cover problem. *Sci. Rep*. **6**, 22307; doi: 10.1038/srep22307 (2016).
